# Antitumor Activity of Axitinib in Lung Carcinoids: A Preclinical Study

**DOI:** 10.3390/cancers15225375

**Published:** 2023-11-12

**Authors:** Alessandra Dicitore, Germano Gaudenzi, Silvia Carra, Maria Celeste Cantone, Monica Oldani, Davide Saronni, Maria Orietta Borghi, Jacopo Grotteschi, Luca Persani, Giovanni Vitale

**Affiliations:** 1 Department of Medical Biotechnology and Translational Medicine, University of Milan, 20122 Milan, Italy; alessandra.dicitore@libero.it (A.D.); davide.saronni1@gmail.com (D.S.); jacopo.grotteschi@gmail.com (J.G.); luca.persani@unimi.it (L.P.); 2 Laboratory of Geriatric and Oncologic Neuroendocrinology Research, IRCCS, Istituto Auxologico Italiano, 20145 Milan, Italy; g.gaudenzi@auxologico.it (G.G.); c.cantone@auxologico.it (M.C.C.); monicaoldani12@gmail.com (M.O.); 3 Laboratory of Endocrine and Metabolic Research, IRCCS, Istituto Auxologico Italiano, 20145 Milan, Italy; s.carra@auxologico.it; 4 Department of Clinical Sciences and Community Health, University of Milan, 20122 Milan, Italy; maria.borghi@unimi.it; 5 Experimental Laboratory of Immuno-Rheumatology, IRCCS, Istituto Auxologico Italiano, 20145 Milan, Italy

**Keywords:** lung carcinoid, tyrosine kinase inhibitors, axitinib, cell cycle, apoptosis, zebrafish, tumor xenograft

## Abstract

**Simple Summary:**

Lung carcinoids (LCs) are categorized as low- and intermediate-grade neuroendocrine tumors. Surgery is effective for localized LCs with a favorable prognosis. However, there is a pressing need for new treatments for unresectable and metastatic LCs. This study explored the potential antitumor effects of axitinib using both in vitro (immortalized cell lines) and in vivo (zebrafish) LC models. Axitinib demonstrated significant antitumor activity by reducing cell viability, inducing cell cycle arrest, and promoting cell death. Moreover, axitinib had a notable impact on crucial processes in tumor progression, including tumor-induced angiogenesis and invasiveness.

**Abstract:**

Lung carcinoids (LCs) comprise well-differentiated neuroendocrine tumors classified as typical (TCs) and atypical (ACs) carcinoids. Unfortunately, curative therapies remain elusive for metastatic LCs, which account for 25–30% of cases. This study evaluated the antitumor activity of axitinib (AXI), a second-generation tyrosine kinase inhibitor selectively targeting vascular endothelial growth factor receptors (VEGFR-1, VEGFR-2, VEGFR-3) in human lung TC (NCI-H727, UMC-11, NCI-H835) and AC (NCI-H720) cell lines. In vitro and in vivo (zebrafish) assays were performed following AXI treatment to gather several read-outs about cell viability, cell cycle, the secretion of proangiogenic factors, apoptosis, tumor-induced angiogenesis and migration. AXI demonstrated relevant antitumor activity in human LC cells, with pronounced effects observed in UMC-11 and NCI-H720, characterized by cell cycle perturbation and apoptosis induction. AXI significantly hindered tumor induced-angiogenesis in *Tg(fli1a:EGFP)^y1^* zebrafish embryos implanted with all LC cell lines and also reduced the invasiveness of NCI-H720 cells, as well as the secretion of several proangiogenic factors. In conclusion, our study provides initial evidence supporting the potential anti-tumor activity of AXI in LC, offering a promising basis for future investigations in mammalian animal models and, eventually, progressing to clinical trials.

## 1. Introduction

Lung carcinoids (LCs) are neuroendocrine tumors (NETs) that comprise well-differentiated typical (TCs) and atypical carcinoids (ACs) classified as low- and intermediate-grade NETs (G1 and G2), respectively [1,2]. Surgical resection represents the mainstay treatment for localized TC and AC. Although LCs are generally less aggressive than other primary lung tumors, approximately 25–30% of patients, especially those with AC, develop distant metastases. Since curative therapies are not available for metastatic forms, there is an urgent need for new treatment options [3,4,5,6,7,8].

LCs express a plethora of growth factors and their relative receptors that can promote tumor progression by stimulating angiogenesis and metastasis formation [9,10]. A high expression of vascular endothelial growth factor (VEGF) has been correlated with a relevant angiogenesis as well as decreased progression-free survival in patients with low-grade NETs, such as LCs [11]. VEGF receptors (VEGFR-1, -2 and -3) are highly expressed in LCs, suggesting that the autocrine activation of the VEGF pathway may promote tumor growth as observed in other NETs [12,13]. Interestingly, it has been demonstrated that VEGR-2 and -3 are more expressed in TC and AC than in other lung cancers [13]. Other tyrosine kinase receptors, such as stem cell factor receptors (c-kit), endothelial growth factor receptor (EGFR), platelet-derived growth factor (PDGFR)-α and, PDGFR-β have been identified in a significant number of patients with metastatic LCs and in LC cell lines [12,14,15,16,17,18].

In light of the heterogeneity in the expression of growth factors and related receptors in LCs, several tyrosine kinase inhibitors (TKIs) have been evaluated for the treatment of LCs [15,19,20,21]. In this context, it is valuable to explore other TKIs that have shown effectiveness in treating different types of tumors as potential candidates for managing LCs.

Axitinib (AXI) is a potent and selective TKI of VEGFR-1, -2 and -3 [22] that received approval by the European Medicines Agency in 2015 for the treatment of advanced renal carcinoma after a failure of prior treatment with sunitinib or interleukin 2 [23]. Moreover, AXI has been tested as second-line therapy in hepatocellular carcinoma and non-small-cell lung cancer [24,25]. An open-label, two-stage design, phase II trial showed an inhibitory effect of AXI on tumor growth in patients with progressive unresectable/metastatic low-to-intermediate-grade NETs [26]. 

In this study, we characterized the antitumor activity of AXI on human lung TC (NCI-H727, UMC-11, NCI-H835) and AC (NCI-H720) cell lines by collecting both in vitro and in vivo read outs.

## 2. Materials and Methods

### 2.1. Drug Preparation and Cell Line Cultures

AXI was provided by MedChemExpress (Monmouth Junction, NJ, USA) and diluted in dimethyl sulfoxide (DMSO) at a concentration of 10^−2^ M. In this study, we used the following four human cell lines, distributed by ATCC: NCI-H727, UMC-11 and NCI-H835, as representative of TC, and NCI-H720 as representative of AC. 

NCI-H727 was derived from a bronchial tumor of a 65-year-old white female smoker. UMC-11 cell line was derived from a lung tumor, but no information about the patient was available. NCI-H835 was derived from a lung tumor of a 48-year-old black nonsmoking female. NCI-H720 was derived from the lung tumor of a male. 

All cell lines were maintained at 37 °C in 5% CO_2_ and cultured in T75 flasks filled with 10 mL of RPMI medium (EuroClone™, Milan, Italy). Medium was supplemented with 10% heat-activated fetal bovine serum (FBS) (EuroClone™, Milan, Italy) and 10^5^ of U·L^−1^ penicillin/streptomycin (EuroClone™, Milan, Italy). Cells were harvested via trypsinization, resuspended in complete medium and then counted through an optical microscope using a standard hemocytometer before plating. Cells used in all experiments were below 5 passages.

### 2.2. Cell Viability Assay 

To assess cellular metabolic activity as an indicator of cell viability, we performed a colorimetric assay, such as tetrazolium salt 3-(4,5-dimethylthiazol-2-yl)-2,5-diphenyltetrazolium bromide (MTT), for the adherent NCI-H727 and UMC-11 cell lines, whereas 3-(4,5-dimethylthiazol-2-yl)-5-(3-carboxymethoxyphenyl)-2-(4-sulfophenyl)-2H-tetrazolium (MTS) assay was performed on NCI-H835 and NCI-H720 cell lines growing in suspension. Human LC cells were seeded in 96-well plates at a density of 6 × 10^3^ cells/well for NCI-H727 and UMC-11; 5 × 10^4^ cells/well for NCI-H835 and 2 × 10^4^ cells/well for NCI-H720. The day after, cell culture medium was replaced with medium containing vehicle (DMSO), or different concentrations of AXI. After 3 days of incubation, cell culture medium was replaced with a fresh one containing vehicle or drug at different concentrations. After 6 days, cells were analyzed using MTT (for NCI-H727 and UMC-11) or MTS (for NCI-H835 and NCI-H720) assays, as previously described [27,28].

### 2.3. Flow Cytometric Analysis of Cell Cycle and Apoptosis

Human lung carcinoid cells were plated in duplicates in 6-well plates at density of: 1 × 10^5^ cells/well for NCI-H727 and UMC-11 and 3 × 10^5^ cells/well for NCI-H835 and NCI-H720. On the following day, the cell culture medium was replaced with medium containing either the vehicle (DMSO) or AXI at EC_50_ concentrations. After 3 days, cells were harvested via gentle trypsinization, washed with cold PBS and collected through centrifugation. Flow cytometry techniques were used to analyze the cell cycle and apoptosis, using propidium iodide (PI) and Annexin V-FITC/propidium iodide staining, respectively. For the cell cycle analysis, a PI (Sigma-Aldrich^®^ Merck KGaA, Darmstadt, Germany) solution (50 μg/mL PI, 0.05% Triton X-100 and 0.6 μg/mL RNase A in 0.1% sodium citrate) was added to stain the pellets at 4 °C for 30 min and cells were analyzed through flow cytometry (FACScalibur, BD Biosciences, San Jose, CA, USA) with a focus on 10,000 events to detect G_0_/G_1_ versus S versus G_2_/M cell percentages. For apoptosis, cells were resuspended in 100 μL of 1× binding buffer (BB: 1.4 M NaCl, 0.1 M HEPES/NaOH, pH 7.4, 25 mM CaCl_2_). After staining with 5 μL of Annexin V-FITC (BD Pharmingen, San Diego, CA, USA) and 10 μL PI (50 μg/mL in PBS) for 15 min at room temperature in the dark, 400 μL of 1× BB was added to each sample and cells were analyzed, as reported before, using FACScalibur flow cytometer on 10,000 events. Moreover, by staining cells with a combination of Annexin-V-fluorescein isothiocyanate (FITC) and PI, it is possible to identify viable cells (Annexin-V−/PI−), early apoptotic cells (Annexin-V+/PI−), late apoptotic cells (Annexin-V+/PI+) and necrotic cells (Annexin-V−/PI+). 

### 2.4. Western Blot Analysis 

Human LC cells were plated onto 6-well plates, following the same procedure described earlier for the flow cytometric analysis of cell cycle and apoptosis. The following day, cell culture medium was replaced with medium containing either the vehicle (control) or AXI at EC_50_ concentrations. After 3 days, cells were scraped, washed twice in cold PBS and resuspended in radioimmunoprecipitation assay (RIPA) lysis buffer, which contained protease and phosphatase inhibitors. 

Lysates were centrifuged at 13,000× *g* for 15 min at 4 °C and protein content in each supernatant was analyzed with BCA protein assay. Ten μg of cell extracts per lane were separated on NuPage 4–12% Bis/Tris Gels and transferred through iBlot System (Life Technologies, Carlsbad, CA, USA). Subsequently, membranes were blocked with 5% milk TBS-T and incubated with specific first antibodies (dilution 1:1000) at 4 °C overnight: anti-caspase-3 (full and cleaved) and anti-Poly (ADP-ribose) polymerase (PARP) (full and cleaved) (Cell Signaling Technology, Beverly, MA, USA). After the incubation, the blots were treated with HRP-conjugated mouse and rabbit secondary antibodies (dilution 1:5000). The blots were then detected using ECL Star Enhanced Chemiluminescent Substrate (EuroClone, Milan, Italy) and the bands were visualized with the Azure Imaging Systems (Azure Biosystems, Dublin, CA, USA). The results were normalized by the level of actin expression in each sample. Intensities of the bands were expressed as arbitrary units when compared to controls.

### 2.5. In Vivo Zebrafish Assays for Tumor-Induced Angiogenesis and Tumor Cell Migration

Embryo and adult zebrafish (*Danio rerio*) were raised and maintained according to Italian (D.Lgs 26/2014) and European laws (2010/63/EU and 86/609/EEC). At 48 h post fertilization (hpf) *Tg(fli1a:EGFP)^y1^* embryos [29] were anesthetized with 0.016% tricaine (ethyl 3-aminobenzoatemethanesulfonate salt, Sigma-Aldrich^®^ Merck KGaA, Darmstadt, Germany) and then placed on agarose-modified Petri dish where they were oriented with the yolk on one side. Subsequently, they were implanted with human LC cell lines, as previously described [30,31]. Briefly, tumor cells were labelled with a red fluorescent viable dye (CellTrackerTM CM-DiI Dye, Invitrogen™ Thermo Fisher Scientific, Waltham, MA, USA) following manufacturer’s instructions. The labelled cells were resuspended in PBS, and then grafted (about 100–1000 cells per embryo) into the sub-peridermal space of *Tg(fli1a: EGFP)^y1^* embryos, close to the sub-intestinal vein (SIV) plexus. The injections were carried out using a micro-injector FemtoJet (Eppendorf, Hamburg, Germany), equipped with a micromanipulator InjectMan NI 2 (Eppendorf, Hamburg, Germany). All implanted embryos were raised at 32 °C, which served as a compromise temperature between the optimal temperature for zebrafish maintenance (28 °C) and the optimal temperature for mammalian cell growth and metabolism (37 °C). After the implantation, zebrafish embryos were exposed to AXI treatment for up to 48 h with AXI being dissolved into fish medium. As control for the pharmacological treatment, we included grafted embryos incubated in the fish water and the vehicle in which the experimental substance was dissolved (DMSO). This transplantable platform was employed to assess the AXI effects on tumor-induced angiogenesis and the spread of tumor cells. The pro-angiogenic and migratory response of injected tumor cells were monitored in vivo by means of an epifluorescence stereomicroscope Leica M205 FA equipped with a digital camera Leica DFC 450 C and using the LAS software (4.2.0 version, Leica Microsystems, Wetzlar, Germany). As an arbitrary unit of tumor-induced angiogenesis at 24 h post injection (hpi), we calculated in each imaged embryo the EGFP area corresponding to endothelial structures that sprouted from the SIV plexus by using Fiji software (version 2.9.0/14 September 2022) [32]. The presence of tumor cell clusters far from the injection site, in the tail region, was detected via fluorescence microscopy and quantified using the “Analyze Particle” tool of Fiji software at 0 (immediately after the injection) and 48 hpi. 

### 2.6. Analyses of Human VEGF, Angiopoietin 1–2, FGF-1α, PDGF (AB, AA, BB, C) Concentrations in Conditioned Media of LC Cell Lines

Human LC cell lines were plated in triplicates in 24-well plates at a density of 1.5 × 10^5^ cells/well. The following day, the cell culture medium was replaced with a medium containing AXI (0.25 µM) or vehicle (DMSO). After 24 h of treatment, cell culture media were collected and stored at −80 °C until analyzed.

The concentrations of human growth factors, such as VEGF (vascular endothelial growth factor, cat#KHG0111), FGF-1α (fibroblast growth factor 1α; cat#EHFGF1), PDGF-AB (platelet-derived growth factor-AB, cat# EHPDGFAB), PDGF-AA (cat#EHPDGFA), PDGF-C (cat#EH365RB), PDGF-BB (cat#BMS2071) and Angiopoietin 1 (cat#EHANGPT1) and 2 (cat#MAN0004094) in the culture medium were quantified using an enzyme-/linked immunosorbent (ELISA) assay by Invitrogen (Invitrogen™ Thermo Fisher Scientific, Waltham, MA, USA), following manufacturer’s instructions. All values were normalized to the cellular proteins in each group.

### 2.7. Statistical Analyses

All in vitro experiments were carried out at least 3 times, providing comparable results. Comparative statistical evaluation among groups was first performed using Analysis of Variance (ANOVA). When significant differences were found, a comparison between groups was made using Newman–Keuls as post hoc test for differences between concentration groups in dose–response curves. Additionally, unpaired Student’s *t* test was performed to analyze statistical differences in cell cycle modulation, apoptosis, relative quantifications of protein expression for Western blotting and data from ELISA assay. A *p* value < 0.05 was considered statistically significant. 

All in vivo experiments were performed at least three times with each experimental group consisting of 20 embryos. Statistical differences among groups were evaluated vis ANOVA test, followed by Tukey’s multiple comparison test as a post hoc analysis. A *p* value < 0.05 was considered statistically significant. 

Values reported in all graphs represented the mean ± standard error of the mean (S.E.M). For all statistical analyses, GraphPad Prism 5.0 (GraphPad Software, San Diego, CA, USA) was used.

## 3. Results

### 3.1. AXI Affected Viability of Human LC Cell Lines

**Figure 1:**
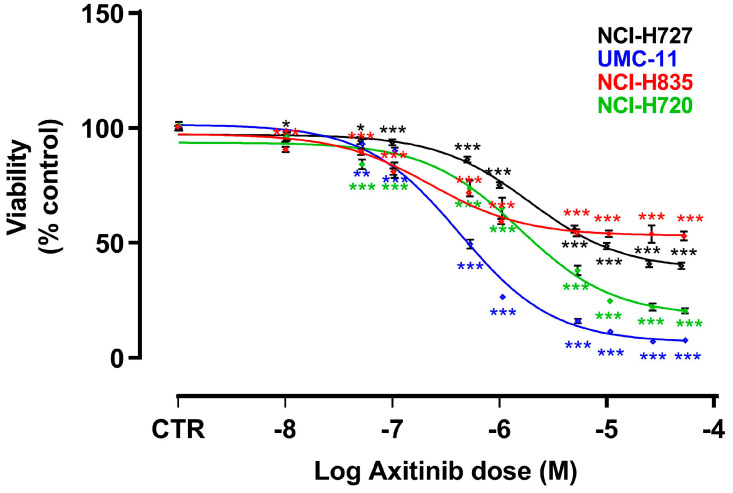
Effects of AXI on cell viability after 6 days of treatment. Dose response curves were generated after MTT (for NCI-H727 and UMC-11) or MTS (for NCI-H835 and NCI-H720) assays and are expressed as nonlinear regression (curve fit) of log (concentration drug) versus the percentage of vehicle control (CTR). Values represent the mean and S.E.M. of at least three independent experiments. * *p* < 0.05; ** *p* < 0.01; *** *p* < 0.001.

In order to analyze the effects of AXI on the viability of four LC cell lines (NCI-H727, UMC-11, NCI-H835 and NCI-H720), MTT or MTS assays were performed after 6 days of incubation. The choice of this time frame was influenced by the low proliferation rate of NET cell lines. AXI significantly decreased the viability of all cell lines in a dose-dependent manner (Figure 1). To compare the effects on different cell lines, EC_50_ and maximal inhibition were calculated. The EC_50_ of AXI was significantly lower in UMC-11 (4.2 × 10^−7^ M) and NCI-H835 (2.2 × 10^−7^ M) compared with that observed in NCI-H720 (1.4 × 10^−6^ M, *p* < 0.001) and NCI-H727 (1.9 × 10^−6^ M, *p* < 0.001) cells. The maximal inhibitory effect was higher in NCI-H720 (−98.2%) and UMC-11 (−89.1%) compared to NCI-H835 (−49.7%; *p* < 0.001) and NCI-H727 (−64.6%; *p* < 0.001) cells. Subsequent analyses on the effects of AXI on cell cycle and cell death were performed using the EC_50_ concentrations.

### 3.2. AXI Perturbed Cell Cycle in Human LC Cell Lines 

**Figure 2:**
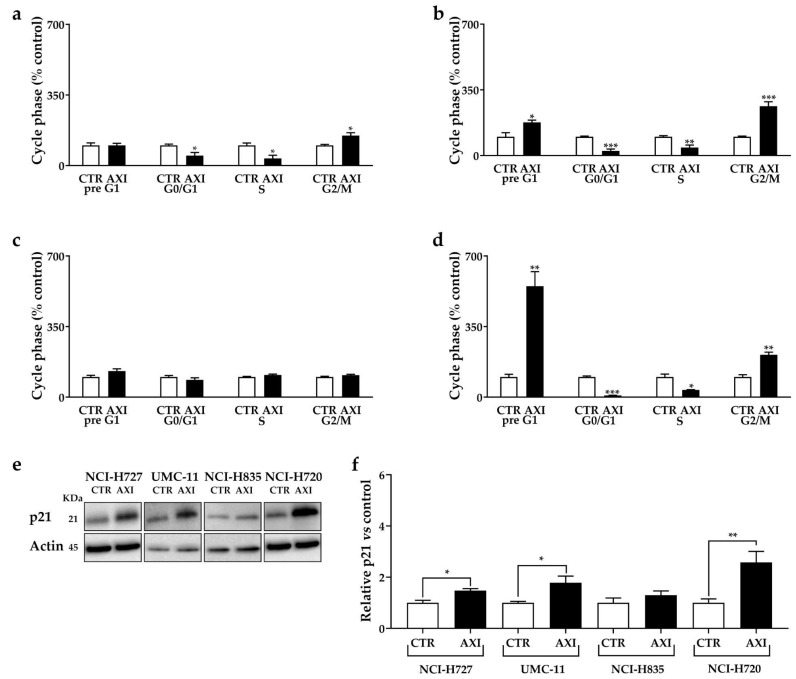
AXI modulation of cell cycle in NCI-H727 (**a**), in UMC-11 (**b**), in NCI-H835 (**c**) and NCI-H720 (**d**) after 3 days of incubation. Cell cycle distribution is expressed as the percentage of cells in G_0_/G_1_, S and G_2_/M phases compared to untreated control cells (CTR). CTR values have been set to 100%. Representative Western blot images (**e**) and relative quantifications (**f**) for the expression of p21 after 3 days of incubation with AXI compared to vehicle control (CTR) in lung carcinoid cell lines. * *p* < 0.05; ** *p* < 0.01; *** *p* < 0.001. The original western blot is Figure S3.

AXI significantly increased the percentage of human LC cells in pre-G_1_ phase (UMC-11: +76%, *p* < 0.05; NCI-H720: +450%, *p* < 0.01), while it decreased the percentage of cells in G_0_/G_1_ (NCI-H727: −50.8%, *p* < 0.05; UMC-11: −75.1%, *p* < 0.001; NCI-H720: −91.2%, *p* < 0.001) and in S phase (NCI-H727: −64.6%, *p* < 0.05; UMC-11: −57.1%, *p* < 0.01; NCI-H720: −64.1%, *p* < 0.05), with a concomitant increase in population in the G_2_/M phase (NCI-H727: +48.4%, *p* < 0.05; UMC-11: +162.9%, *p* < 0.001; NCI-H720: +109.8%, *p* < 0.01) compared to controls (Figure 2a–d). 

Furthermore, we examined the expression of p21 (WAF1/Cip1) protein, an inhibitor of cyclin-dependent kinases and a key regulator of G_1_/S and G_2_/M transitions. AXI increased the p21 expression in NCI-H727, UMC-11 and NCI-H720 cells (Figure 2e,f). No significant perturbation of the cell cycle and variation in p21 expression were observed in NCI-H835 cells after incubation with AXI (Figure 2).

### 3.3. AXI Induced Cell Death in Human LC Cell Lines

**Figure 3:**
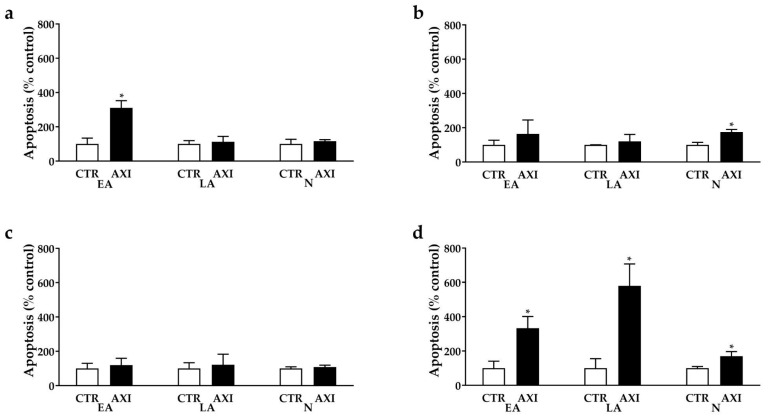
AXI perturbation of cell death in NCI-H727 (**a**), in UMC-11 (**b**), in NCI-H835 (**c**) and NCI-H720 (**d**) after 3 days of incubation. The proportions of early apoptotic cells (EA) and late apoptotic (LA) and necrotic cells (N) are expressed as percentage compared with the untreated control (CTR) and represent the mean and S.E.M. of at least 3 independent experiments. * *p* < 0.05.

Cell death perturbation occurred in NCI-H727, UMC-11 and NCI-H720 cells after incubation with AXI compared with control. AXI increased the percentages of NCI-H727 (+271.5%, *p* < 0.05) and NCI-H720 (+366%, *p* < 0.05) cells in early apoptosis (Figure 3a,d, respectively) and the fraction of NCI-H720 cells in late apoptosis (+480%, *p* < 0.05, Figure 3d) compared to untreated control. In addition, this drug increased the number of UMC-11 (+74.7%, *p* < 0.05, Figure 3b) and NCI-H720 cells (+69.7%, *p* < 0.05, Figure 3d) in necrosis compared to the control. No statistically significant effects on apoptosis were observed in NCI-H835 cells after AXI treatments (Figure 3c). 

**Figure 4:**
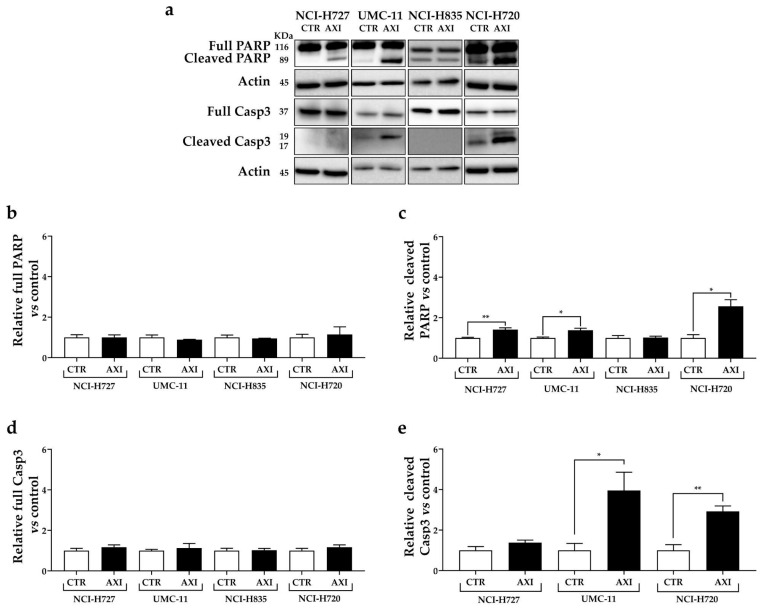
Representative Western blot images (**a**) and relative quantifications for the expression of total (**b**) and cleaved PARP (**c**), total (**d**) and cleaved caspase 3 (**e**) after 3 days of incubation with AXI compared to vehicle control (CTR) in NCI-H727, UMC-11, NCI-H835 and NCI-H720 cells. Actin was used as a loading control. The intensity of each band was expressed as the ratio between the relative intensities of the bands associated with actin. Values represent means ± S.E.M from at least 3 independent experiments. * *p* < 0.05; ** *p* < 0.01. The original western blot is Figure S4.

Interestingly, AXI increased the expression of cleaved PARP in all cell lines, except for NCI-H835, compared to untreated control (Figure 4a,c). Moreover, AXI treatment significantly induced the expression of cleaved caspase-3 in UMC-11 and NCI-H720 cell lines (Figure 4a,e). Otherwise, no statistically significant effects were observed in the expression level of full PARP and full caspase-3 after AXI treatment in all cell lines (Figure 4b,d).

### 3.4. AXI Effects on Tumor-Induced Angiogenesis and Tumor Cell Migration

In order to analyze the effects of AXI treatments on tumor-induced angiogenesis and tumor cell spread, we took advantage of an in vivo platform that we have recently developed for implanting LC cells in *Tg(fli1a:EGFP)^y1^* zebrafish embryos [33]. This zebrafish line expresses EGFP (enhanced green fluorescent protein) under the control of the endothelial-specific gene promoter fli1a, enabling the visualization of the entire vascular network. 

At 48 hpf, zebrafish embryos were implanted with TC (NCI-H835, UMC-11 and NCI-H727) and AC (NCI-H720) cell lines and then treated for 24 and 48 h with AXI. Due to the permeability of embryonic tissue to small molecules, AXI was directly dissolved in the fish medium. Two different concentrations were tested, 0.25 and 2.5 µM, based on preliminary pharmacological experiments on *Tg(fli1a:EGFP)^y1^* embryos without tumor xenograft, aimed at detecting the toxicity range of the drug and minimizing the occurrence of morphological abnormalities. 

**Figure 5:**
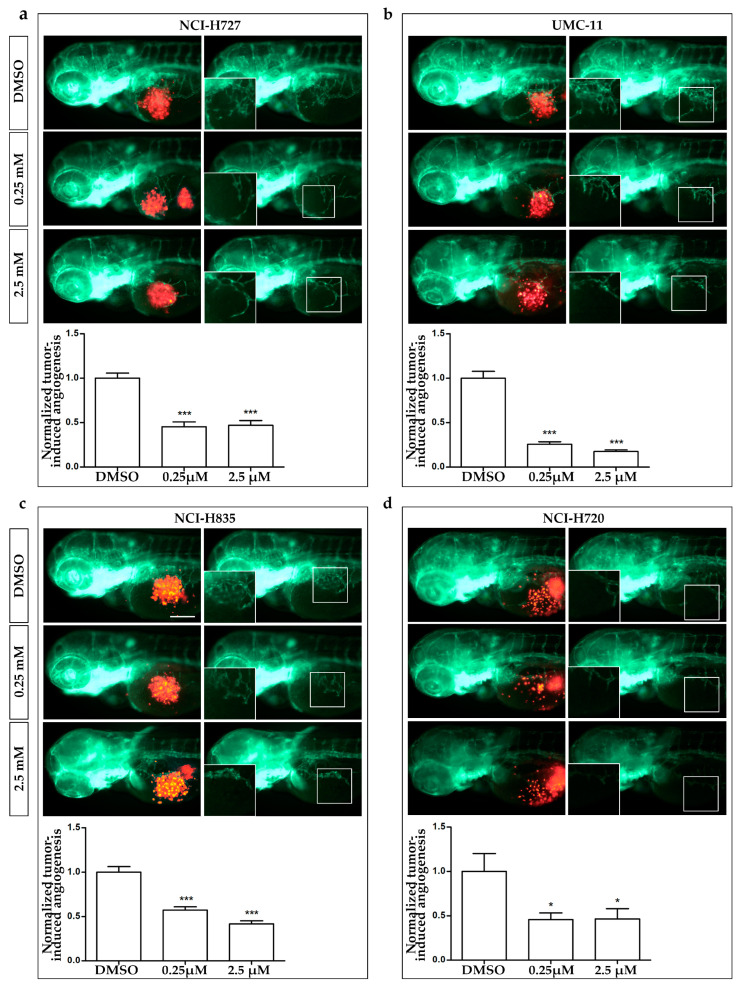
Effects of AXI treatments on tumor-induced angiogenesis in zebrafish embryos implanted with lung carcinoid cells. Representative epifluorescence images of *Tg(fli1a:EGFP)^y1^* zebrafish embryos at 24 hpi, implanted with NCI-H727 (**a**), UMC-11 (**b**), NCI-H835 (**c**) and NCI-H720 (**d**) cells and treated with DMSO, as control, and 0.25 and 2.5 µM AXI. The red channel, corresponding to lung carcinoid cells, was omitted in the second column of each panel to highlight the tumor-induced microvascular network. Digital magnifications of graft regions are shown in white-boxed regions. Embryos are shown anterior to the left. Scale bar, 100 µm. In the bottom of each panel, the graph showed the quantification of tumor-induced angiogenesis in embryos implanted with lung carcinoid cells after 24 h of AXI treatment at 0.25 and 2.5 µM. Control (DMSO) values have been set to 1.0. Graphed values represent the mean ± S.E.M. * *p* < 0.05; *** *p* < 0.001.

**Figure 6:**
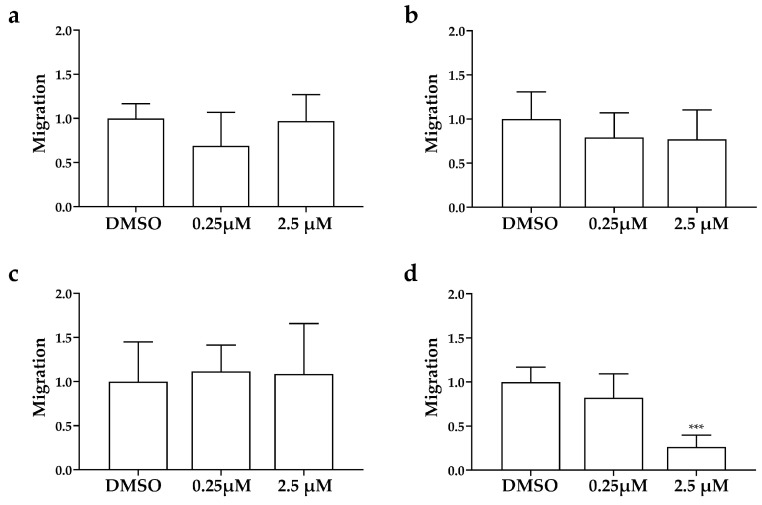
Effects of AXI on invasiveness of human LC cells in grafted zebrafish embryos. Quantification of cell spread in the tail of embryos injected with NCI-H727 (**a**), UMC-11 (**b**), NCI-H835 (**c**) and NCI-H720 (**d**) cells at 0 and 48 hpi after treatment with AXI (0.25 and 2.5 µM). As arbitrary unit of migration we considered the number of fluorescent particles in the tail. Graphed values represent the mean ± S.E.M. *** *p* < 0.001.

After only 24 h of treatment (Figure 5), AXI strongly inhibited tumor-induced angiogenesis in *Tg(fli1a:EGFP)^y1^* embryos injected with all LC cells compared to controls. This effect was particularly pronounced in embryos injected with UMC-11 cells (Figure 5b). In addition, AXI markedly reduced tumor cell invasiveness in zebrafish embryos implanted with NCI-H720, as evidenced by the significant decrease in fluorescent cells along the tail after 48 h of treatment with the highest concentration of AXI compared to controls (Figure 6d). Otherwise, no statistically significant effects were observed in the invasiveness of the other cell lines after AXI (Figure 6a–c).

### 3.5. AXI Modulated Human VEGF, Angiopoietin 1–2, FGF-1α, PDGF Concentrations in Conditioned Media of LC Cell Lines

**Figure 7:**
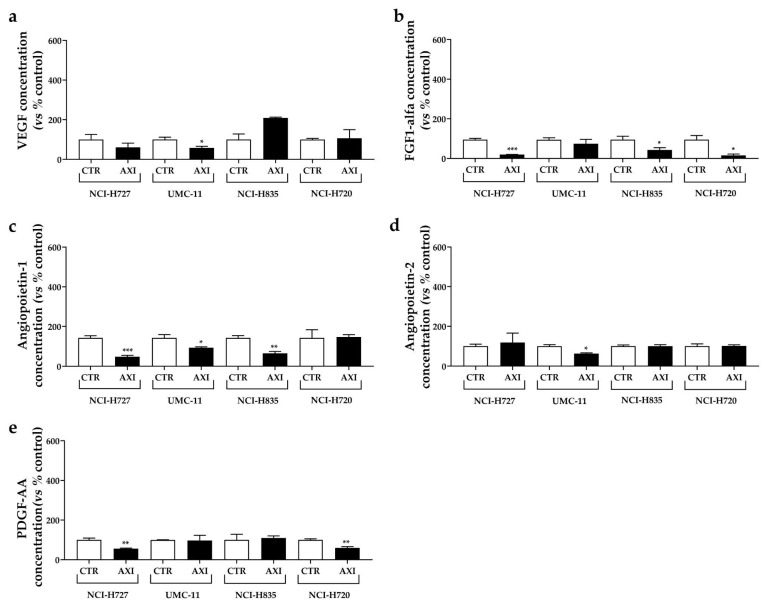
Effects of AXI incubation on the production of pro-angiogenic growth factors in human LC cell lines. VEGF (**a**), FGF-1α (**b**), Angiopoietin-1 (**c**), Angiopoietin-2 (**d**) and PDGF-AA (**e**) were measured via ELISA on cell culture media after 24 h of AXI incubation in LC cell line, as discussed in Materials and Methods. All values were normalized to the cellular proteins of each group. Results were expressed as a percentage compared with the vehicle-treated control (CTR) and represent the mean and S.E.M. of at least three independent experiments. * *p* < 0.05; ** *p* < 0.01; *** *p* < 0.001.

Considering the significant inhibition of tumor-induced angiogenesis and tumor cell invasiveness observed in vivo after 24 h of treatment with AXI, we assessed the concentrations of several growth factors involved in these pathways. In particular, conditioned media from LC cell lines were collected and analyzed after 24 h of incubation with or without 0.25 µM AXI to simulate in vivo conditions (Figure 7a–e).

Following AXI treatment, a significant reduction in VEGF (Figure 7a) and Angiopoietin-2 concentrations (Figure 7d) was observed in conditioned medium from UMC-11 cells (−42.2% and −37.4%, respectively, both *p* < 0.05) compared to control. AXI also decreased FGF-1 secretion (Figure 7b) in two TC cell lines (NCI-H727: −79.4%, *p* < 0.001; NCI-H835: −54.7%, *p* < 0.05) and AT cell line (NCI-H720: −83.9%, *p* < 0.05). Concentrations of Angiopoietin-1 significantly decreased in conditioned medium from TC cell lines (NCI-H727: −67%, *p* < 0.001; UMC-11: −34.6%, *p* < 0.05; NCI-H835: −54.8%, *p* < 0.01) after incubation with AXI compared to control (Figure 7c), while PDGF-AA decreased in NCI-H727 and NCI-H720 cells (−44.3% and −40.3%, respectively, both *p* < 0.01, Figure 7e). 

## 4. Discussion

Surgical resection is the treatment of choice for localized LCs. Patients with distant metastases represent a significant treatment challenge due to their high resistance to radiotherapy and chemotherapy. Current treatments are not curative and primarily aim to manage symptoms arising from the tumor burden or hormonal production, as well as to slow down tumor progression [4,5,6,8,34,35]. In this context, there is an urgent need for the development of novel therapeutic strategies. 

AXI is a potent, selective second-generation inhibitor of VEGFRs, approved in many countries as a second-line treatment of advanced renal-cell carcinoma [22,23]. An open-label, two-stage design, phase II trial of AXI in patients with progressive unresectable/metastatic low-to-intermediate-grade NETs showed an inhibitory effect of AXI on tumor growth. In this study, only 3 out of 30 enrolled patients presented LCs [26]. Thus far, few data have been published about the anti-tumor activity of AXI in LC preclinical models. Wang and co-workers showed that AXI significantly inhibited cell growth in both 2D and 3D culture models of NCI-H727 after 6 days of incubation [36].

In the present preclinical study, we combined in vitro and in vivo approaches to evaluate the antitumor activity of AXI on a broad number of commercially available LC cell lines, including three human TC (NCI-H727, UMC-11 and NCI-H835) and one AC cell line (NCI-H720). It is important to note that while immortalized cell lines may not fully recapitulate the complexity and heterogeneity of LCs, obtaining primary cultures of these neoplasms is challenging due to several factors, including small tissue samples, the low proliferation rate of tumor cells, and the rarity of LC.

In our in vitro experiments AXI was able to significantly decrease the viability rate of all human LC cell lines after 6 days of incubation, especially in UMC-11 and NCI-H720 cells. 

Furthermore, the antitumor activity of this TKI was modulated by the induction of apoptosis/necrosis and cell cycle arrest. Nowadays, no data are available about the effects of this compound on cell cycle and apoptosis in lung NETs.

In two TC (NCI-H727, UMC-11) and AC (NCI-H720) cell lines, AXI significantly decreased the percentages of cells in G_0_/G_1_ and S phase and promoted the accumulation of cells in G_2_/M phase. This effect was extremely relevant in UMC-11 and NCI-H720 cells. A cell cycle perturbation of LC cells after incubation with AXI has also been confirmed by the increased expression of p21Waf1/Cip1, a protein that controls cell cycle progression and negatively regulates cellular proliferation, being involved in p53-dependent G_1_ and G_2_ cell cycle arrest [37,38]. Moreover, AXI exerted a prominent pro-apoptotic activity in LC cell lines, especially in NCI-H720, as detected via staining with Annexin V/PI and analyzed via FACS, and increased caspase-3 activation and PARP cleavage. The cleaved PARP-1 (89 kDa, C-terminal fragment) is indeed considered a signal of a point of no return in the late-stage of apoptosis [39]. Notably, when cells encounter genomic instability due to DNA damage, PARP-1 initiates the repair machinery. However, if the extent of DNA damage exceeds the cell’s repair capacity, PARP-1 is cleaved by caspases-3. Despite the limitation of the use of immortalized cell lines, future examinations can be beneficial to better understand cell death mechanisms induced by this TKI in LC. Specifically, further investigations should focus on the NCI-H835 cell line, which exhibited a higher resistance compared to other cell lines. 

NETs are highly vascularized neoplasms. Several studies reported that well-differentiated NETs are hyper-vascularized with respect to poorly differentiated neuroendocrine carcinomas [40,41,42,43]. In this context, high vascular density is more likely to be a marker of differentiation than a marker of aggressiveness, as usually appearing in other carcinomas. This phenomenon, named as the “neuro-endocrine paradox”, has been mainly described in gastroenteropancreatic NETs, but it has also been reported in lung NETs [44,45]. Indeed, it has been described that serum levels of hepatocyte growth factor (HGF), VEGF and VEGFR-1 were significantly higher in patients with carcinoid tumors versus the healthy control group, and only VEGFR-1 levels closely correlated with TNM classification [46]. Mairinger and co-workers demonstrated that VEGR-2 and -3, as well as c-fos-induced growth factory, an activator of VEGFRs, were more expressed in TC and AC than in other lung NETs [13]. 

In order to further investigate the antitumor activity of AXI, we analyzed in vivo its effects on tumor-induced angiogenesis and tumor cell dissemination, exploiting the procedure of the tumor xenotransplantation in zebrafish embryos. While murine models are widely regarded as the gold standard for tumor xenografts, zebrafish embryos emerged as a valuable alternative model due to their peculiar advantages and their adherence to the principles of the 3Rs. Zebrafish transparency during embryonic stages allows for a real-time observation of tumor growth and progression, tumor–host interactions, tumor-induced angiogenesis and migration. Tumor implantation in zebrafish embryos can overcome some relevant drawbacks reported in mice. For instance, the maintenance costs of a zebrafish facility are lower compared to those of mice, and their management is straightforward. The response to tumor implantation in zebrafish embryos, in terms of proangiogenic effects of implanted cells or their metastatic behavior, can be readily observed in real-time and only after 24 hpi, a time window narrower than that required in mice, ranging from weeks to months. Immunosuppression is not required because zebrafish embryos do not possess a fully developed immune system, preventing graft rejection at this stage of development. Furthermore, zebrafish offer the possibility to study the effects of small tumor implants (100–1000 cells/embryo) compared to the larger implants (about 1 million cells) required in murine models [47]. Another crucial aspect to emphasize is the conservation of tumor-induced angiogenesis between zebrafish and humans in terms of key signaling pathways and molecular players, such as VEGF and its receptors. Studies using zebrafish have demonstrated its utility in evaluating the effectiveness of anti-angiogenic drugs and identifying potential therapeutic agents for inhibiting tumor-induced angiogenesis. The results obtained from zebrafish experiments often correlate with those from human studies, underscoring the predictive value of this model organism in cancer research [48]. Furthermore, tumor xenografts in zebrafish embryos provide a valuable platform to perform the drug screening of new anticancer molecules. Due to the permeability of zebrafish embryos to small molecules (i.e., TKI), these drugs can be directly added to the embryo water. This makes zebrafish transplantable models suitable for the preliminary high-throughput screening of anti-cancer drugs, which can help reduce costs before moving on to more expensive mammalian models. While tumor xenografts in zebrafish embryos offer several advantages, it is important to acknowledge certain potential limitations. For instance, zebrafish embryos are maintained at 28 °C, which may not provide the optimal temperature for the growth and metabolism of mammalian cells. Additionally, the absence of some mammalian organs in fish, such as the mammary gland, prostate, and lung, restricts the possibility of performing orthotopic transplantations as seen in mouse models. Furthermore, although embryonic organs and systems are well-defined, their differentiation remains incomplete in embryos. This, coupled with physiological differences between fish and mammals, may influence drug metabolism in zebrafish, potentially leading to variations compared to mammalian systems [47]. Regarding LCs, we have recently [33] provided evidence of the validity of zebrafish embryo xenotransplantation for studying angiogenesis and migration in this group of tumors. Interestingly, we observed a strong tumor-induced angiogenesis in embryos implanted with TC cell lines, with respect to those implanted with AC cells, which displayed a higher ability to spread along the embryo body. These data confirmed the neuroendocrine paradox in this preclinical model and strongly suggested that the zebrafish model faithfully recapitulates the tumor behavior of LCs in humans [33]. 

We exploited our zebrafish platform to assess the effectiveness of AXI, focusing on its anti-angiogenic and anti-migratory properties. Within just 24 h, AXI strongly inhibited tumor-induced angiogenesis, particularly in embryos injected with UMC-11 cells. This result is intriguing, considering our in vitro observations of a remarkable inhibition of UMC-11 viability after AXI exposure. The heightened sensitivity of UMC-11 cells to AXI treatment, compared to other LC cell lines, may be attributed to their distinct pattern of secreted factors. Notably, UMC-11 cells were found to secrete the highest concentration of VEGF among LC cells, as quantified in the conditioned medium via the ELISA test (Figure S1), suggesting a pivotal role of the VEGF system in their survival. Interestingly, AXI treatment led to a significant reduction in the secretion of several pro-angiogenic factors (VEGF, FGF-1, PDGF-AA, Angiopoietin 1 and 2) in LC cell lines after 24 h of incubation. However, VEGF secretion was significantly inhibited only in UMC-11 cells after AXI (Figure 7a). In this context, AXI could be particularly effective in UMC-11 cells by inhibiting both VEGFR activation and VEGF secretion.

Moreover, it is noteworthy that AXI inhibited tumor cell invasiveness only in zebrafish embryos implanted with NCI-H720. 

With respect to the AXI concentrations used in our experiments, it is worth noting that they fall within the same order of magnitude as the reported Cmax in patients with advanced renal-cell carcinoma (27.8 ng/mL, corresponding to 0.72 × 10^−7^ M) [49]. Specifically, in the MTT and MTS assays, we observed that AXI concentrations, similar to the reported Cmax, significantly and moderately inhibited cell viability. Notably, in the context of tumor-induced angiogenesis, AXI concentrations near the Cmax demonstrated a more substantial effect, leading to a reduction in tumor-induced angiogenesis of up to 50% (Figure S2). Axitinib is generally well-tolerated at these concentrations, with a safety profile expected for this class of agents. Common adverse events include hypertension, fatigue, and diarrhea [49].

## 5. Conclusions

AXI exhibited a strong anti-proliferative effect in lung TC and AC cell lines through cell cycle perturbation and pro-apoptotic activity. Additionally, it demonstrated a significant anti-angiogenic potential. In summary, our study has yielded initial evidence of AXI’s potential anti-tumor effects in LC. These findings provide a promising basis for future research in experimental mammalian models of LC and, eventually, progressing to clinical trials. 

## Data Availability

The data presented in this study are available from the corresponding author on request.

## Supplementary Materials

The following supporting information can be downloaded at: https://www.mdpi.com/article/10.3390/cancers15225375/s1, Figure S1: VEGF-1 quantification; Figure S2: tumor-induce angiogenesis at Cmax; Figure S3: The original western blot of Figure 2; Figure S4: The original western blot of Figure 4.
