# Supplementary material for: Antitumor Activity of Axitinib in Lung Carcinoids: A Preclinical Study
Source: Cancers (Basel). 2023 Nov 12;15(22):5375. doi: 10.3390/cancers15225375 (PMC10669991; doi:10.3390/cancers15225375)

P21 **figure S3**

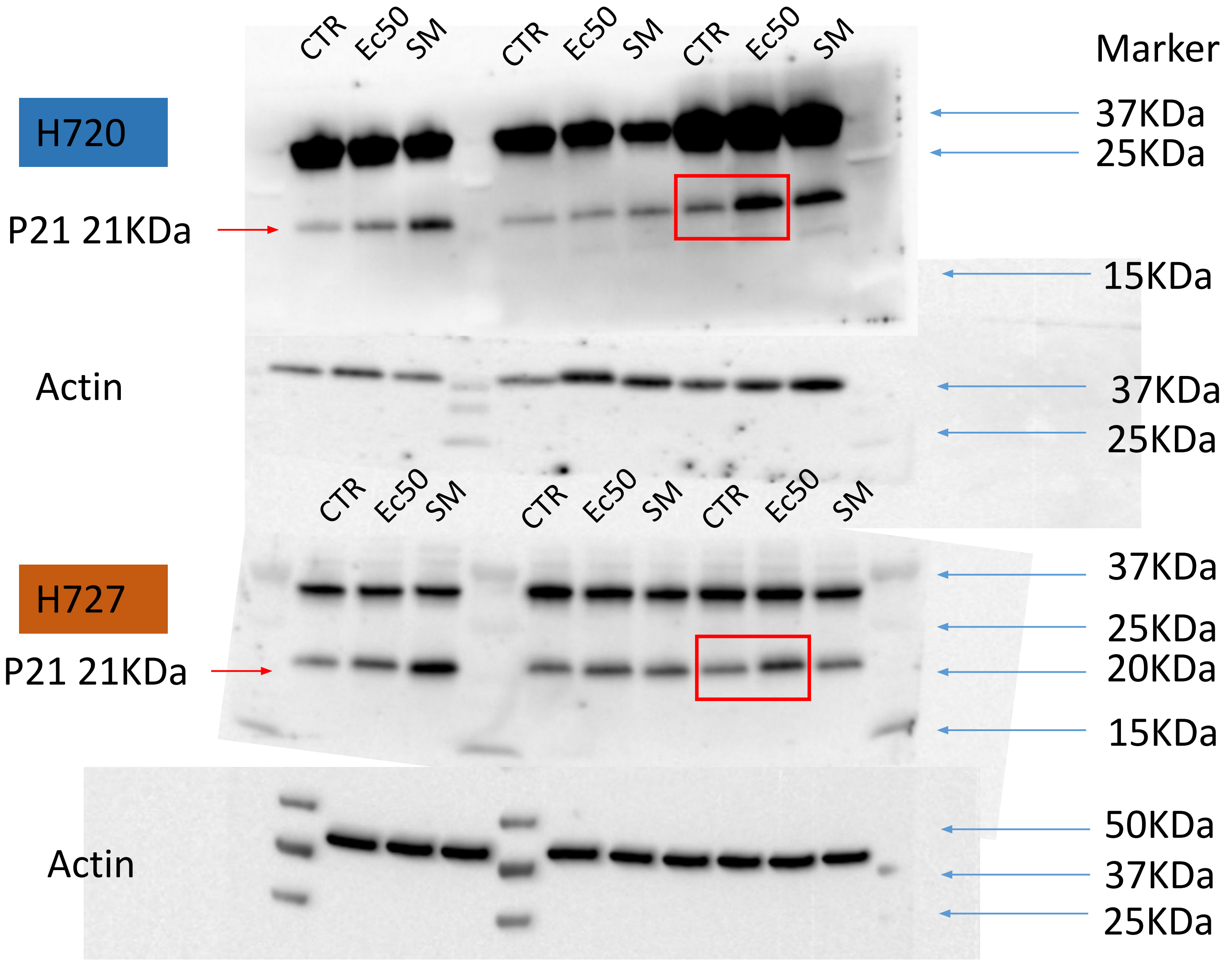

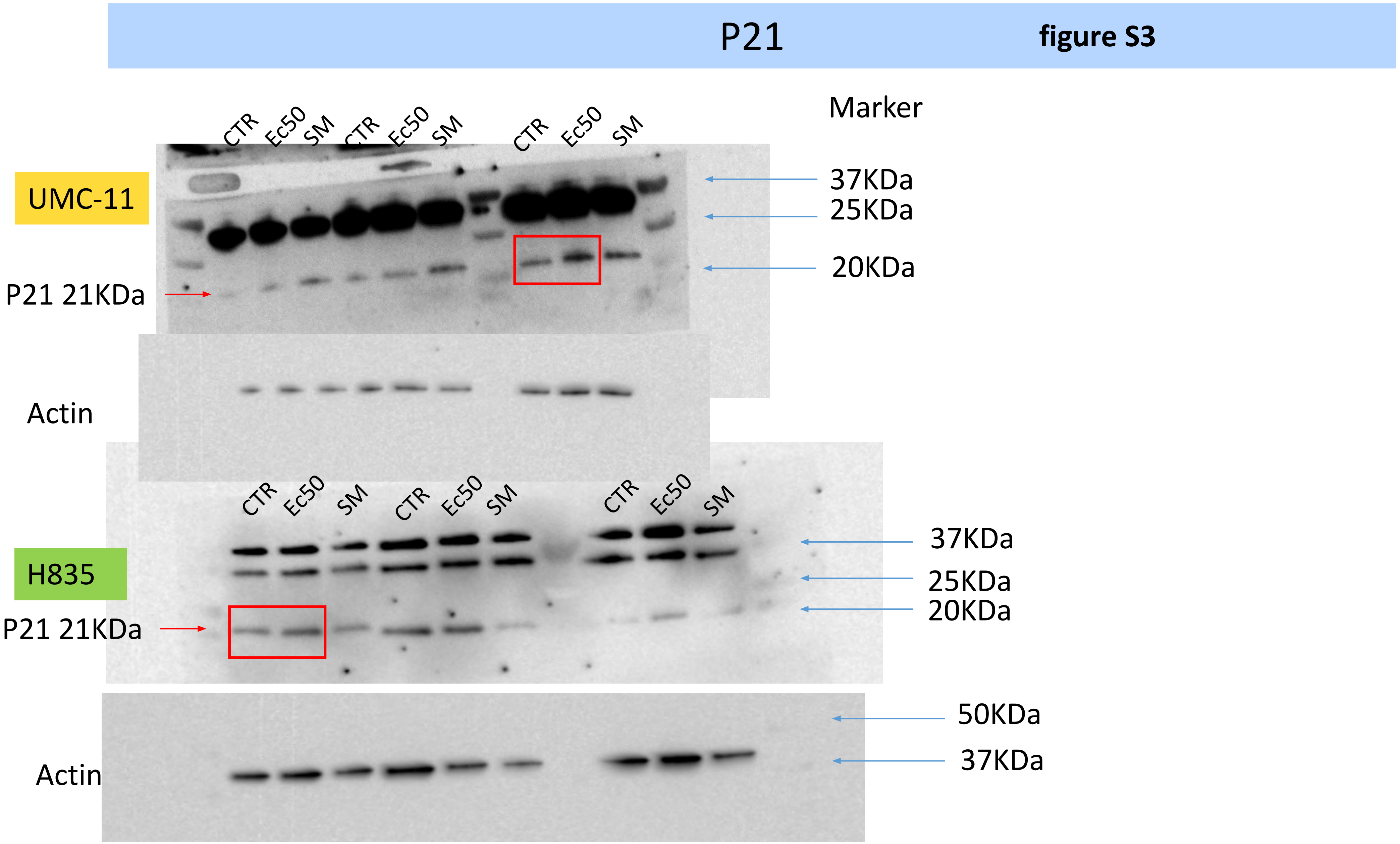
P21
figure S3
Marker
CTR
Ec50
SM
CTR
Ec50
SM
CTR
Ec50
SM
UMC-11
37KDa
25KDa
20KDa
P21 21KDa
Actin
CTR
Ec50
SM
CTR
Ec50
SM
CTR
Ec50
SM
H835
37KDa
25KDa
20KDa
P21 21KDa
Actin
50KDa
37KDa

Cleaved and full Casp3 **figure S4**

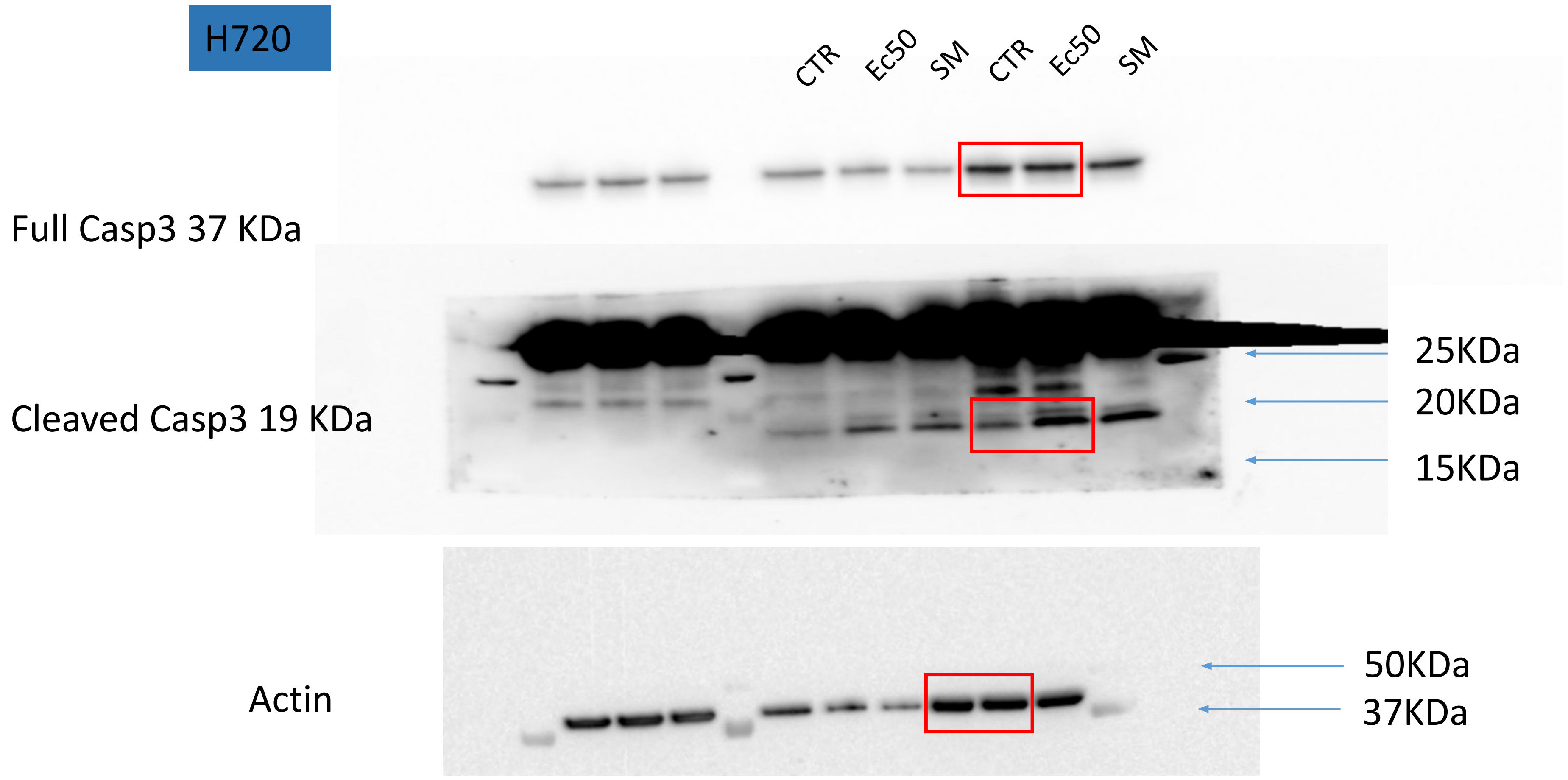

Cleaved and full Casp3

figure S4

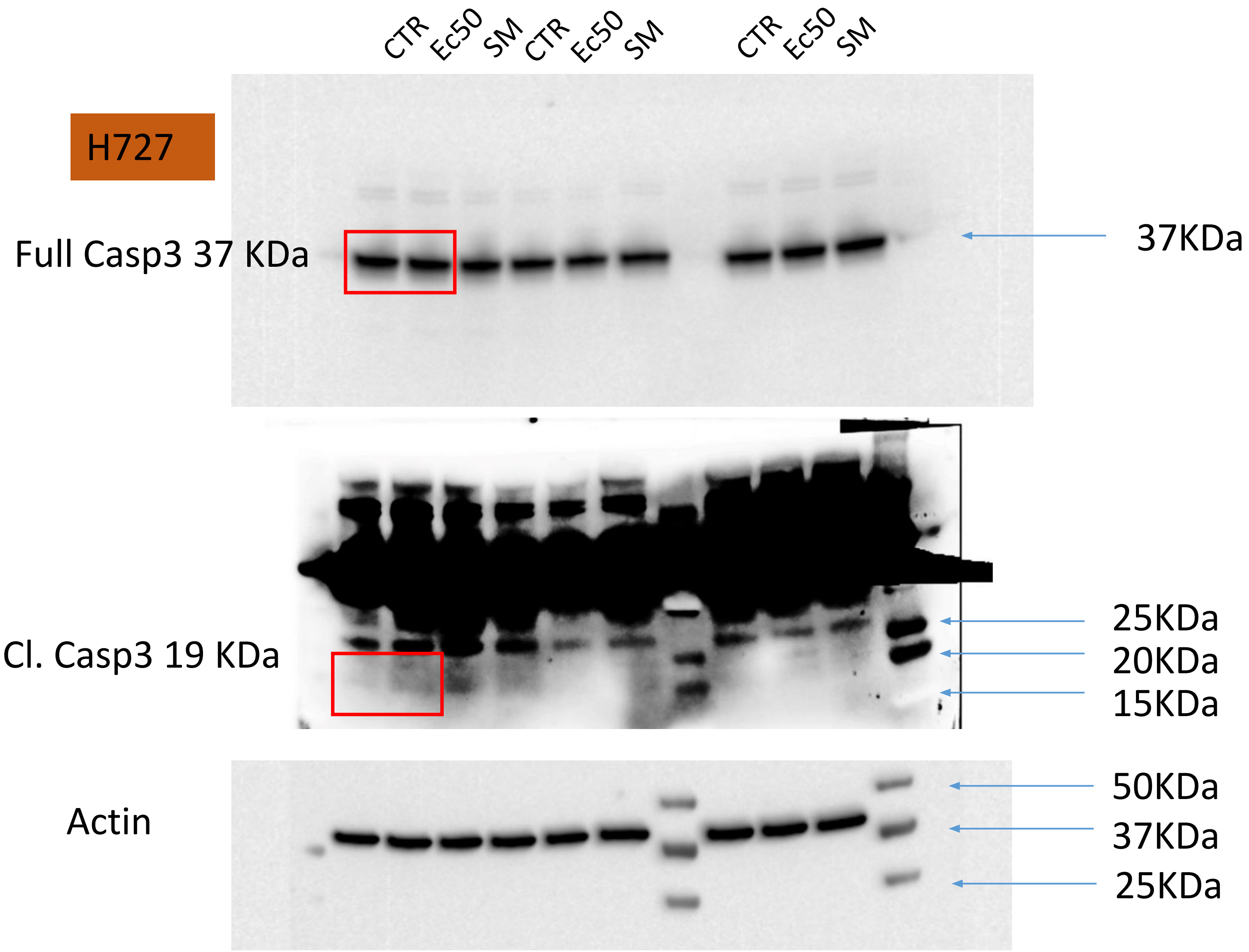

Cleaved and full Casp3

figure S4

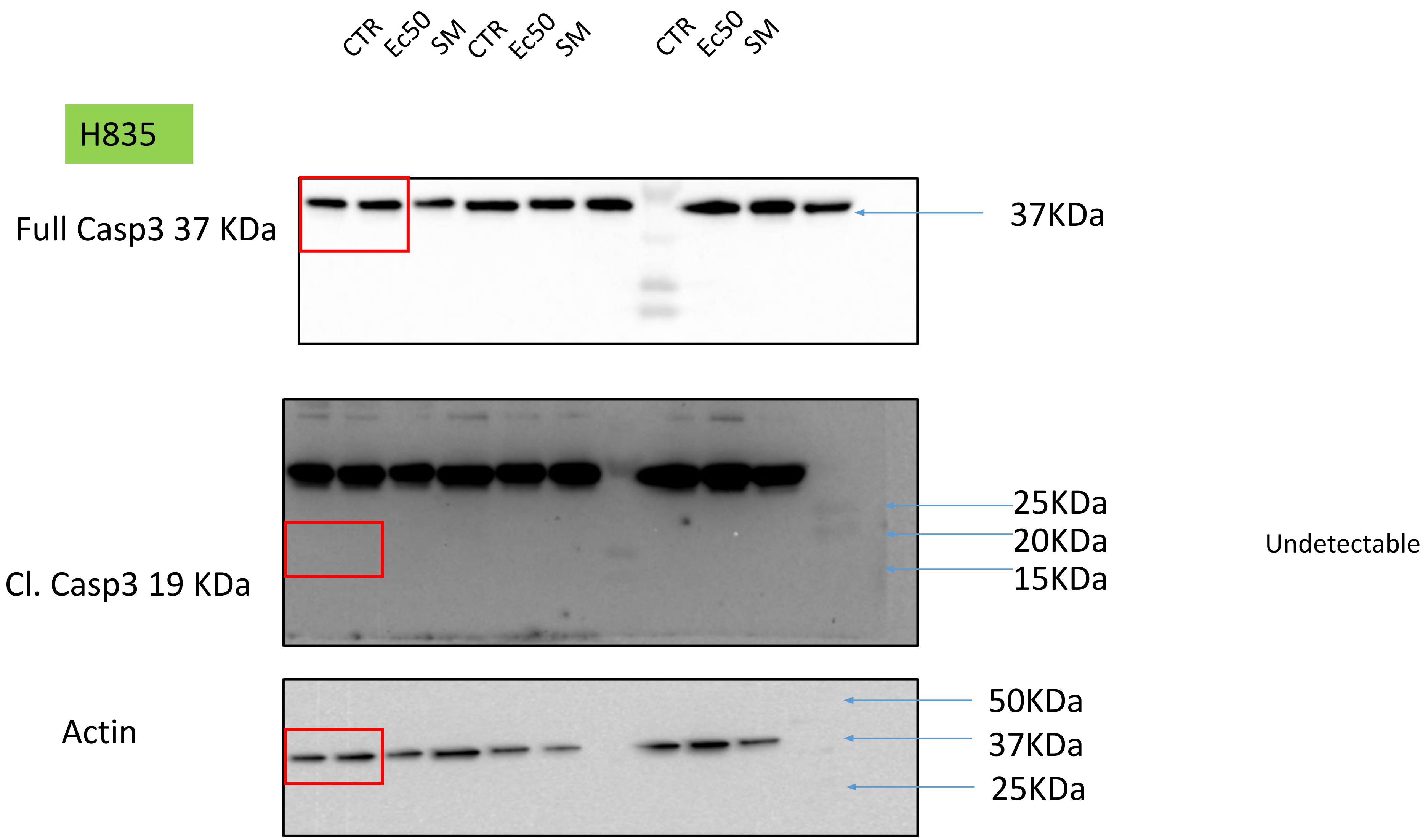

Cleaved and full Casp3 **figure S4**

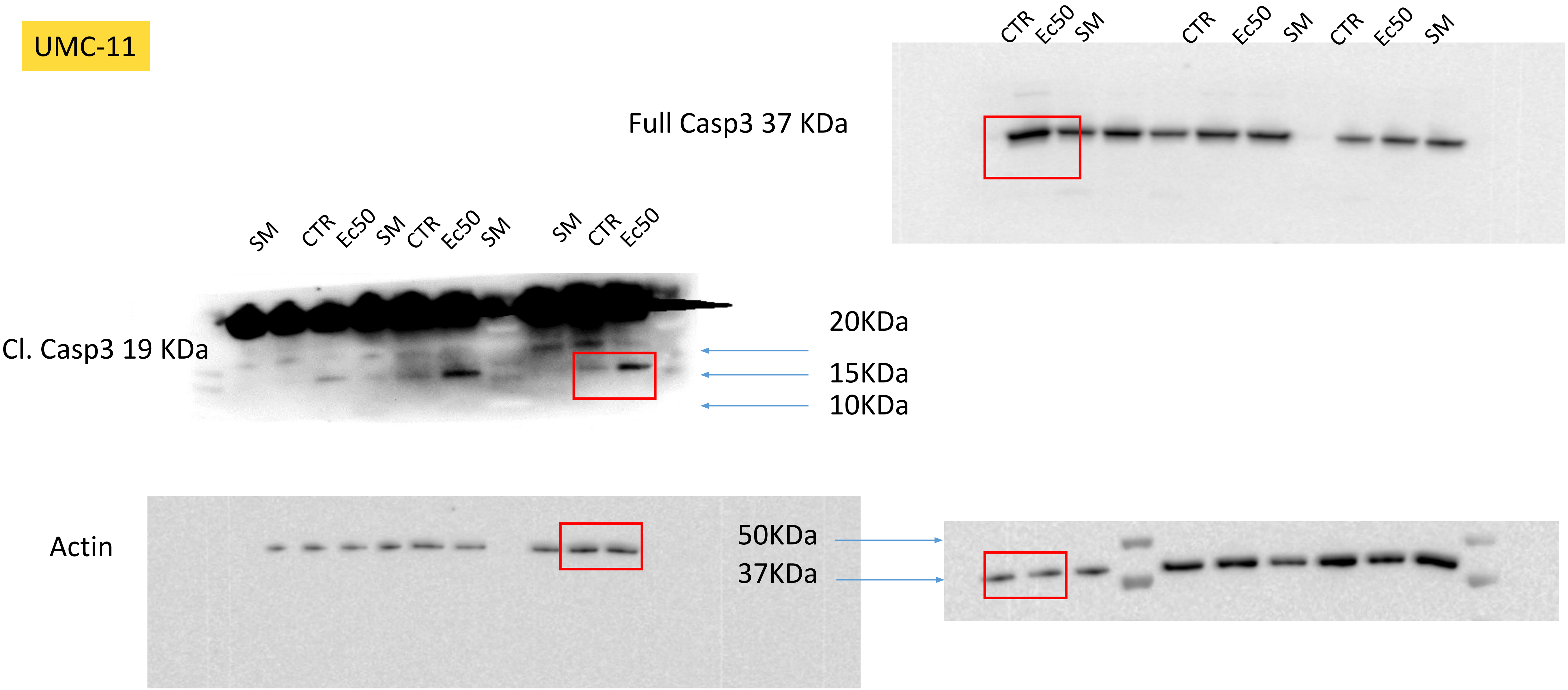

Cleaved and full PARP-1

figure S4

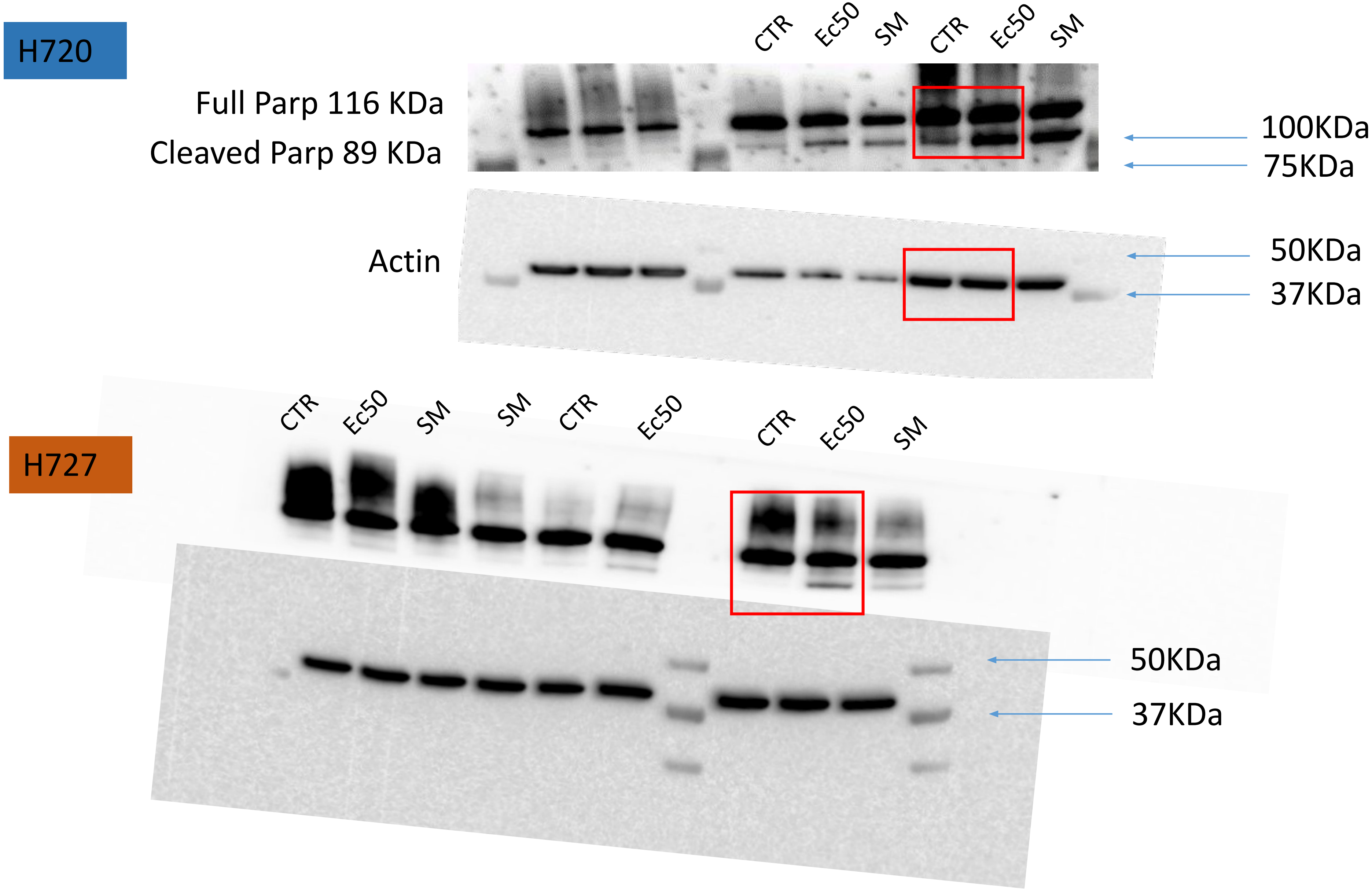

Cleaved and full PARP-1

figure S4

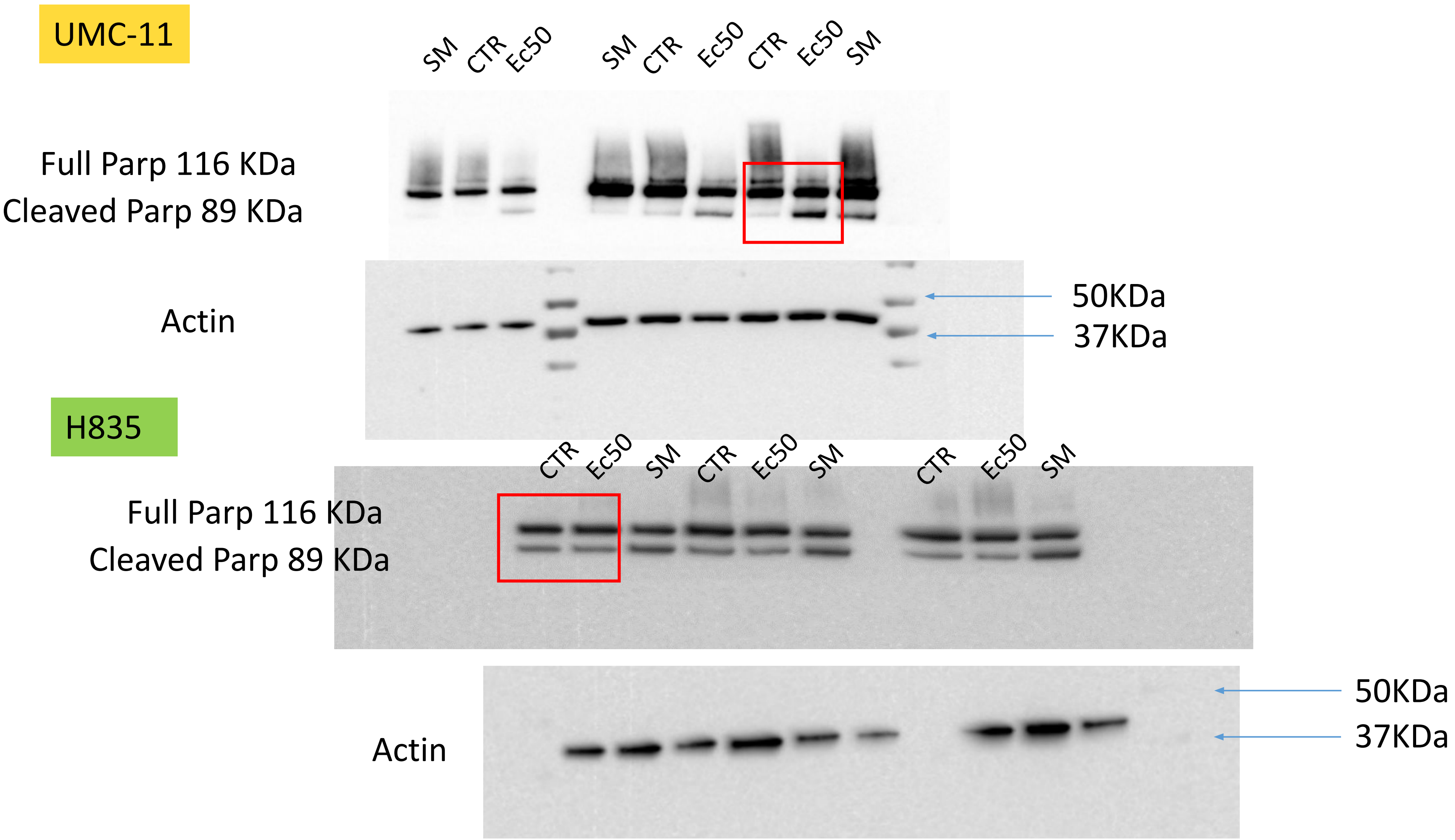

Supplement: Supplementary file 1 [file cancers-15-05375-s001.zip › cancers-2597834-western blot.pdf]
