# Supplementary figures and images for: Antitumor Activity of Axitinib in Lung Carcinoids: A Preclinical Study
Source: Cancers (Basel). 2023 Nov 12;15(22):5375. doi: 10.3390/cancers15225375 (PMC10669991; doi:10.3390/cancers15225375)

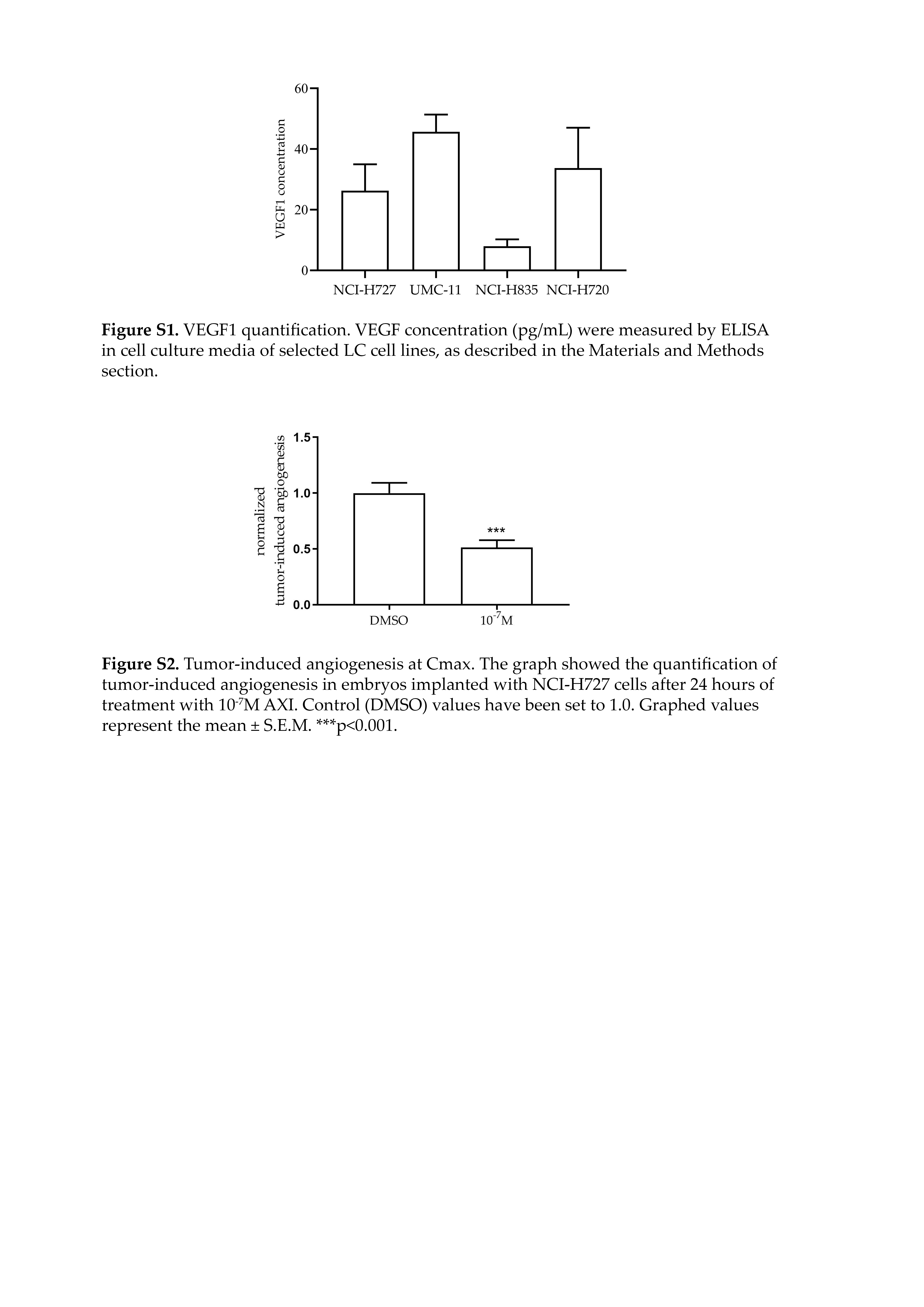

Supplement: Supplementary file 1 [file cancers-15-05375-s001.zip › cancers-2597834-supplementary figures.jpg]
